# The Caveats of observing Inter-Trial Phase-Coherence in Cognitive Neuroscience

**DOI:** 10.1038/s41598-018-20423-z

**Published:** 2018-02-14

**Authors:** Rosanne Maria van Diepen, Ali Mazaheri

**Affiliations:** 1 Department of Psychiatry, Academic Medical Center, University of Amsterdam, Amsterdam, Netherlands; 2 School of Psychology, University of Birmingham, Birmingham, United Kingdom

## Abstract

Many studies have now consistently reported that the phase angle of ongoing oscillatory activity (measured using EEG/MEG), at time of stimulus presentation influences detection when stimuli are near-threshold. However, studies examining whether the adjustment of the phase angle of oscillations is under top-down attentional control have thus far yielded conflicting results. A possible source for the discrepancy could be that the estimation of the phase of ongoing oscillations as well as its uniformity across trials could be affected by task induced changes in the power of oscillations or concurrent evoked responses. One measure, Inter-Trial Phase-Locking (ITPC), or the uniformity of phase angles across trials, is particularly vulnerable to these factors. Here, using various simulations modelling the common task induced changes in the EEG reported in the literature, we demonstrate that apparent changes in Inter-Trial Phase-Locking of oscillatory activity can occur independent of any actual change in the phase of the ongoing activity.

## Introduction

EEG and MEG signal contains oscillatory (i.e rhythmic) activity in various frequency bands. The most predominant oscillatory activity in the EEG/MEG is at the alpha rhythm (8–12 Hz), which has been observed primarily in the sensory systems (vision, motor, auditory). A number of studies have reported that the amplitude of alpha activity is suppressed in sensory relevant regions during visual, auditory and somatosensory attention, but increased in regions responsible for processing unattended information^1–8^. This has led to the speculation that an increase in the amplitude of alpha range oscillations plays a mechanistic role in cognition by gating information flow to relevant sensory regions through the inhibition of irrelevant regions^9–11^.

In addition to the amplitude of alpha activity, several studies have recently found evidence that the alpha cycle reflects states of low and high excitability which has an influence (depending on the arrival of the stimulus in the cycle) on perception^12–15^ as well as on the evoked response to sensory stimuli^16^. Recently, an intriguing possibility put forward has been that top-down processes such as attention and expectation can modulate the phase of alpha activity as a mechanism to either select and prioritize relevant information or conversely suppress information^17^.

However, empirical support for this hypothesis has not been consistent. One study supporting this theory has found that the phase of the ongoing alpha-activity, measured using EEG, appeared to be perturbed by temporal expectation of predictable visual targets^18^. Specifically, the authors observed that cues signalling the arrival of predictable visual stimuli appeared to shift the phase of ongoing alpha activity to be at an ‘optimal’ state at the arrival of the visual stimuli.

On the other hand, our group, using a similar cued temporal expectation paradigm was unable to find any evidence for the modulation of the phase of the ongoing alpha activity in three independent EEG experiments^7^. One critical difference between our experiments was that we conducted our phase-analysis trials on ‘blank’ catch trials that did not include the presentation of a visual stimulus, as such free from sensory evoked responses^7^. As we will demonstrate in the coming sections of this simulation study, these transient evoked responses, which are phase-locked to the onset of sensory stimuli, can critically confound the phase-estimation of ongoing oscillatory activity.

The endeavor of this simulation study is to demonstrate some of the caveats faced by researchers in quantifying event related phase-perturbations (i.e the degree of phase modulation as a result of an experimental event) that might be able to explain the divergence in results. Quantification of event-related phase modulations is commonly reported using Inter-Trial Phase-Locking (ITPC or Inter-Trial-Coherence/Phase-Locking Factor)^18^. An ITPC value close to 0 reflects high variability of phase angles across epochs, whereas an ITPC value of 1 reflects all epochs having the same phase angle.

Here we will first investigate how ITPC estimates can be distorted by modulation of evoked responses. Differences in amplitude or latency of evoked responses can lead to differences in ITPC values that are not actually reflecting phase-perturbation of the ongoing oscillations. Next, we will investigate how the amplitude of the oscillation relative to the noise could modulate ITPC. This endeavor is of particular relevance to investigations of phase perturbations in which there is a systematic difference in the power of oscillatory activity between conditions.

## Influence of ERP amplitude and latency on pre-stimulus ITPC

The onset of a visual stimulus elicits several evoked responses at the scalp, with C1 being the earliest, occurring 50–80 ms after the stimulus. The amplitude of the C1 is generally thought to be unaffected by top-down factors such as attention^19,20^, although some evidence seems to suggest this not to be unequivocal^21^. Following the C1 components are the P1 (~90–140 ms) and the N1 (~150–180 ms) components whose amplitudes have been found in number to be modulated by top-down factors such as attention and expectation^22–31^. Furthermore, several studies have suggested that the P1 and N1 may reflect different components of attention with the P1 reflecting inhibition and the N1 amplification of sensory input^22,32,33^. Finally, it should be noted that responses as early as 27 ms post-stimulus have been found in the early visual cortices of the Macaque monkey^19^.

**Figure 1:**
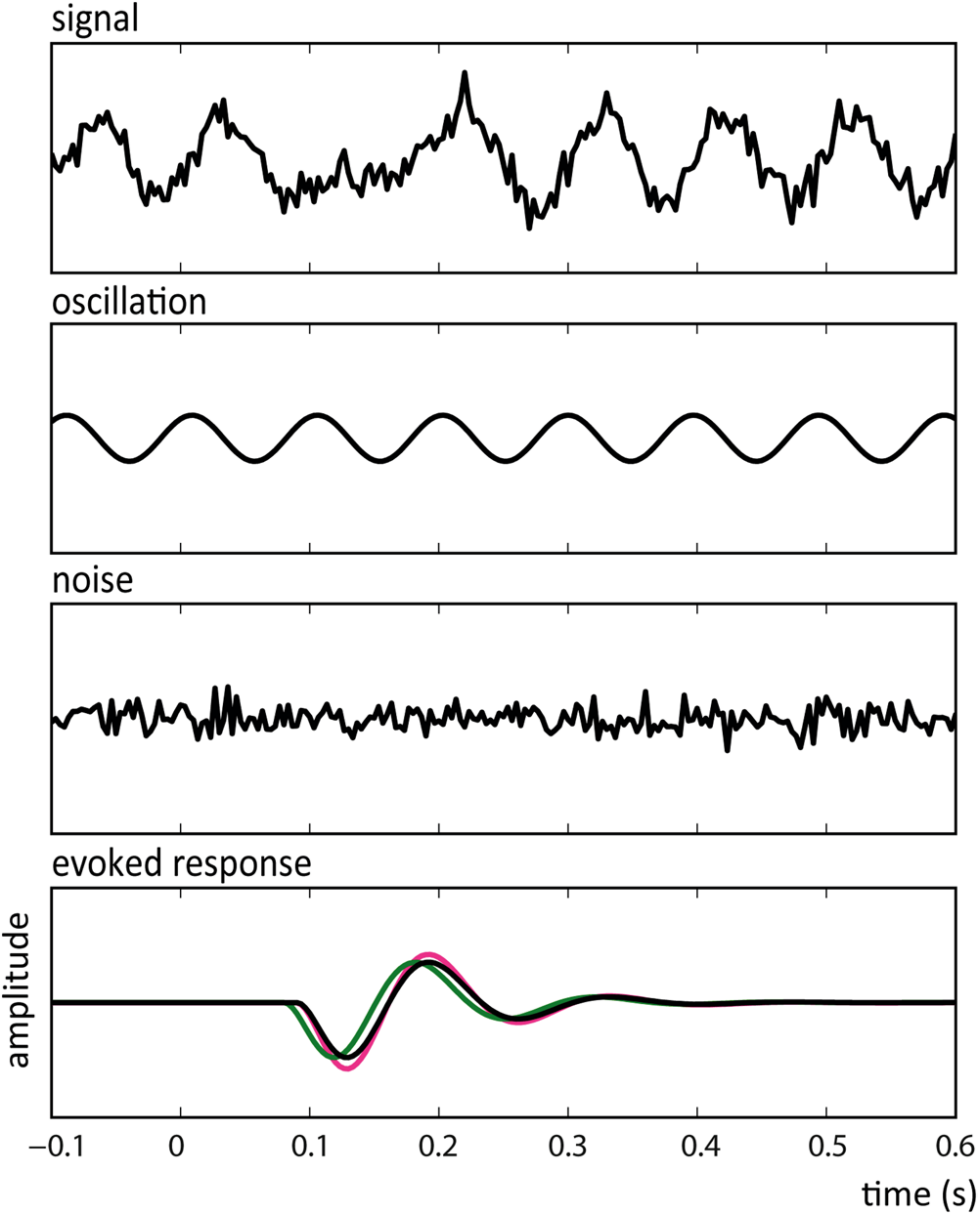
Example of simulated epoch (**A**). Every epoch consisted of (**B**) a sinusoid with a frequency of ~10 Hz and a random phase (**C**) white Gaussian noise and (**D**) an ERP component. Epochs with a ‘standard’ ERP (black line) were compared to epochs with an earlier peak latency (green line) and larger amplitude (pink line).

Temporal leakage from evoked responses containing condition related differences in latency or amplitude could therefore lead to ITPC differences that are actually not present in the ongoing oscillations. Comparing ITPC between conditions is therefore troublesome around the time of stimulus presentation. A simulation was performed in Matlab (MATLAB R2014a, The MathWorks, Inc., Natick, Massachusetts, United States) to demonstrate this phenomenon. One hundred EEG epochs were created by superposition of an ERP component, together with Gaussian white noise, on an alpha oscillation (see Fig. 1).

The ERP component was generated using the following formula:

1 $$ERP(t)=A\frac{t-{t}_{0}}{\tau }{e}^{1-(t-{t}_{0})/\tau }\,\sin \,(2\pi f(t-{t}_{0}))$$

where A is the amplitude of the ERP in μV, *f* is the frequency in Hz, *t* is the time in ms, *t*0 is the time of the start of the ERP and *τ* the exponential decay time of the ERP envelope. For the simulation the frequency of the ERP was 7 Hz, the start of the ERP was at *t* = 90 ms, and *τ* = 50 ms.

The ongoing alpha oscillation was created using:

2 $${\rm{O}}\,(t)={\rm{A}}\,\sin (2\pi \,{\rm{ft}}+\theta )$$

where A is the amplitude of the signal in μV, *f* is the frequency in Hz, *t* is the time in ms and *θ* is the phase in radians. The frequency of the ongoing oscillation was 10 Hz +/− a random scalar drawn from the standard normal distribution (0.5 * randn in Matlab) and the phase was random every trial.

These ‘standard’ epochs were compared with epochs containing an enlarged ERP (1.2 × the standard amplitude) and epochs with a 10 ms earlier peak latency (t = 80 ms versus t = 90 ms). Note that the same noise pattern and phase angles were used for the ‘standard’, ‘earlier’ and ‘larger’ ERP epochs, which means that ITPC should be similar for all three datasets. The phase angle was calculated for every epoch at t = 0 ms (‘target presentation’) by applying a Hann window of 500 ms, taking the Fourier transform and dividing the outcome by its absolute. ITPC was indexed by summation of phase angles of all epochs according to the following formula:

3 $$ITPC({f}_{o},t)=\frac{1}{N}|\sum _{k=1}^{N}{e}^{i{\varphi }^{k}({f}_{o},t)}$$

where N is the number of trials, *φk * is the local phase angle of the signal in the current trial in radians.

**Figure 2:**
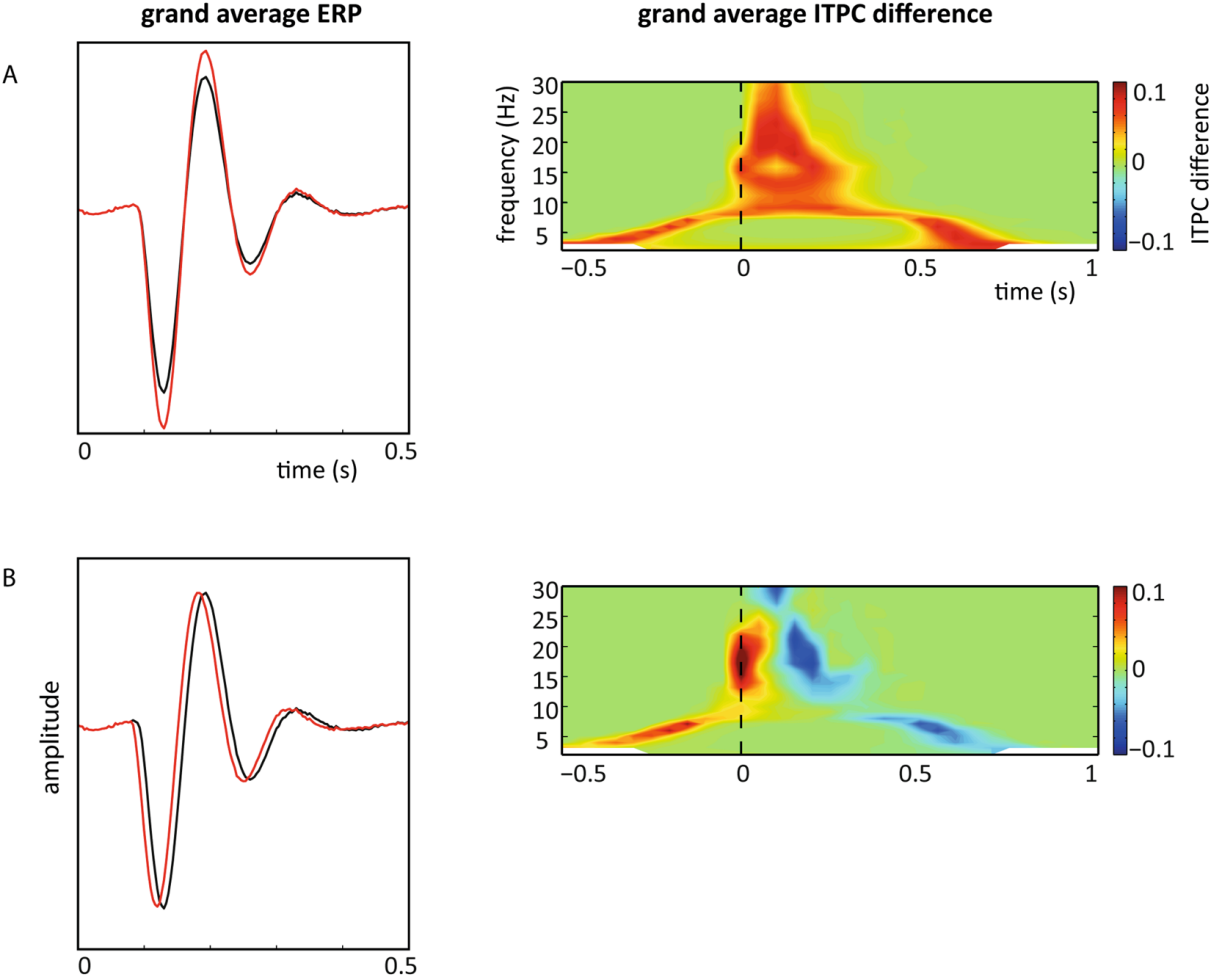
A small increase in amplitude (**A**) or reduction in latency (**B**) of an ERP caused an increase in ITPC around target presentation. The left panels show the grand average ERP of 20 simulated subjects. The right panels show the accompanying grand average of the difference in ITPC between the two ERP conditions.

Here the simulation of 20 participants with 100 epochs per condition, comparing the ‘standard’ ERP to simulations with a larger ERP, led to a difference in ITPC around target presentation (Fig. 2A). A dependent samples t-test revealed that ITPC of 10 Hz oscillations was significantly higher at target presentation for the epochs with the larger ERP (t (19) = −32.3183, p = 4.5150 × 10^−18^) (0.2970 vs 0.2593). Comparing standard epochs with epochs having an earlier peak latency also resulted in a significant difference in ITPC around target presentation (dependent samples t-test (t(19) = −3.2602, p = 0.0014 with higher ITPC for epochs with an earlier ERP peak latency (0.2908 vs 0.2593) (Fig. 2B). To infer how common the ERP induced ITPC difference is, the procedure was repeated 1000 times and the number of times a significant difference was present at 10 Hz was counted. An ERP latency difference between conditions resulted in 80.4% of simulations in a significant difference at target presentation. A difference in amplitude resulted in a significant difference in ITPC between conditions for all simulations (100%).

## Entrainment and phase-locking

Entrainment of oscillatory activity has been suggested as a mechanism for which the brain prepares for processing of temporally predictable targets, (for instance Lakatos *et al*.^34^). The underlying premise here is that the entrainment adjusts the phase-angle of an oscillation such that target presentation falls within an optimal phase. However it should be be kept in mind that a transient top-down signal can also cause a modulation of ITPC in the absence of a real phase modulation. Support for this pitfall comes from Yeung *et al*.^35^ showing that phasic activity cannot be separated from a phase-reset. Similarly, transient activity prior to target presentation can prompt an increase in ITPC when it is time-locked to temporally predictable stimuli. This transient activity does not need to be in the same frequency as the ITPC is measured in. A burst of spiking activity in a high frequency can induce slow ERF’s or ERP’s when oscillations are asymmetrical (i.e. peaks are more positive than troughs are negative^36^), thereby increasing ITPC in lower frequencies. Temporal leakage will then introduce ITPC at later times than the burst actually occurs.

## The influence of oscillatory power on ITPC

**Figure 3:**
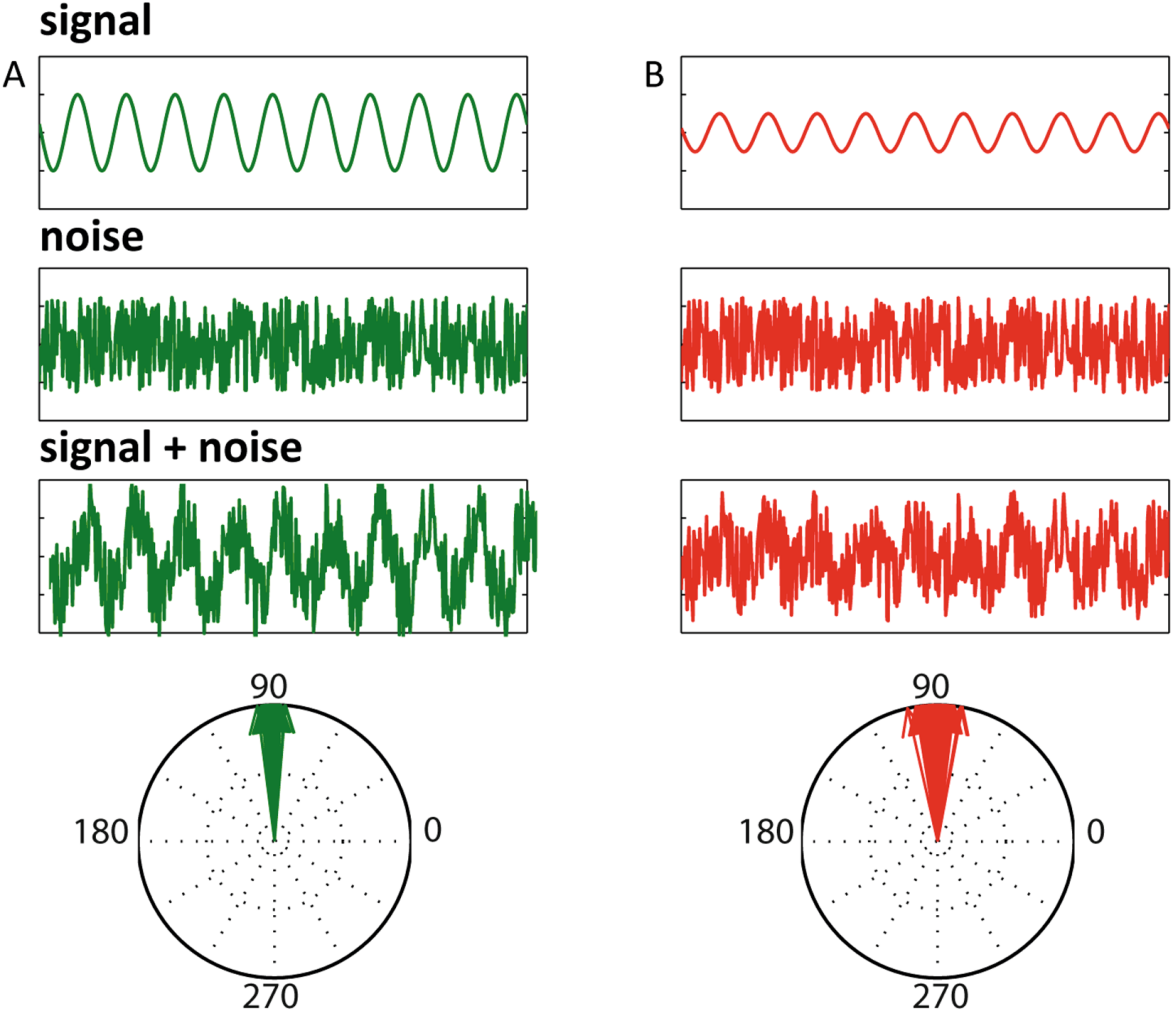
Single epoch with a high power (**A**) and low power (**B**) oscillation. The upper boxes (signal) display the pure sine waves. Middle boxes (noise) is the noise added to both sine waves. Please note that the noise added to both sine waves is identical. Lower boxes (signal + noise) show the simulated EEG epochs which consisted of noise superimposed on the low and high power sine waves. Lower graphs show phase estimations for the low and high power epochs. Although the phase angles and the noise were identical in both conditions, the difference in power introduced a small difference in spread around the true phase angle (ITPC should be 1 in both conditions i.e. complete uniformity across trials).

Power (=amplitude ²) and phase angle are mathematically independent variables. That is, the phase of an oscillation does not depend on its amplitude and vice versa. Both calculations comprise the calculation of the Fourier transform (ft), but in order to obtain the amplitude one must take the absolute value, whereas for the phase angle the argument must be taken. EEG consists of a phasic signal plus noise and a change in signal amplitude will influence the signal to noise ratio (SNR). If noise levels are constant, the SNR is lower for epochs with lower amplitude, making it harder to estimate the phase angle. A difference in power could therefore lead to an ITPC difference; that is the variability in phase angles across epochs at a certain point in time. Given that oscillatory power can differ between experimental conditions, phase estimations will be more precise in one condition than the other. Thus, this could lead to SNR dependent differences in ITPC instead of experimental dependent differences between conditions. Figure 3 shows an example in which differences in the clustering of phase angles emerges simply due to an oscillatory power difference between the two sets of epochs. Both conditions contain a 10 Hz oscillation with ITPC of 1, however for the low power condition ITPC was estimated at 0.9966 and for the high power the estimation was.0.9992. Although, this difference does not appear large, a systematic difference in ITPC caused by a systematic difference in power could eventually lead to a significant difference in ITPC between conditions.

To demonstrate under what conditions power induced ITPC differences can arise, the comparison of ITPC between low and high power 10 Hz oscillations was repeated under different amounts of SNR, ITPC and power. ITPC was calculated over 100 trials for a ‘high’ and a ‘low’ alpha amplitude condition and compared between 20 simulated subjects. Except for the difference in power, everything was kept constant between conditions: that means that the same noise patterns and random phase were added to both the low and high power condition. This results in a well-controlled experiment in which ITPC differences between conditions can only be attributed to power differences.

Given that these simulations are designed to show the influence of noise on phase estimations, the type of noise was chosen to resemble EEG noise as much as possible. Instead of white noise we used noise created with the power spectrum of real EEG data using the implementation of Rafal Bogacz and Nick Yeung (Princeton University, December 2002). No noise in the frequency of our signal of interest (10 Hz) was used, so that the phase angle of the 10 Hz oscillation was completely controlled. The simulations were repeated under different amounts of SNR. SNR levels from −100 to 100 dB (in relation to high power oscillation) were investigated by scaling the noise according to the following steps: The noise was normalized to zero mean and unit variance. Then the following formula was applied:

4 $${\rm{SN}}=\frac{\frac{\sigma \,(\text{signal})}{\sqrt{{10}^{\frac{SNR}{10}}\,}}}{\sigma \,(\text{noise})\,}\cdot {\rm{noise}}$$

where signal is the high power oscillation and SNR the desired signal to noise ratio.

Phase angle and ITPC were calculated at time = 0 ms (centre of the epoch) for the low and high power condition for each subject, as described in paragraph 2 using formula (3). ITPC values were then compared between conditions using an independent samples t-test. This procedure was repeated 100 times to ensure differences were not found by chance.

### Small power difference can lead to a significant difference in estimated ITPC

In the first simulation the difference in power was varied systematically to examine to what extend the power needs to differ between conditions in order to obtain ITPC differences. There the high power condition alpha activity had a fixed amplitude of A = 3 µV and was compared to consecutively low powered conditions in which in which the amplitude was decreased from 0.1 µV steps. Jitter was added to the amplitudes of both conditions by adding a random scalar drawn from the standard normal distribution (0.5 * randn in Matlab). The simulated epochs had an average ITPC of 0.51 (sd = 0.04).

**Figure 4:**
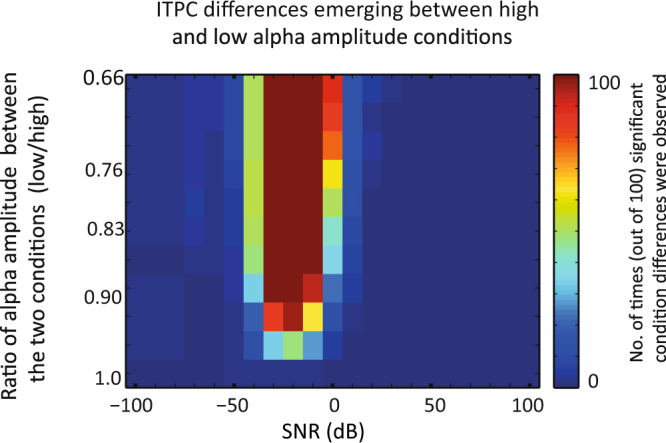
Comparison of ITPC between high and low power epochs for various differences in amplitude. Increasing values on the y-axis represent larger differences in power between conditions. The high amplitude condition had a fixed amplitude of A = 3 µV, while amplitudes for the low power condition decreased from A = 3 µV to A = 2 µV with steps of 0.1. Colors represent the percentage of simulations that resulted in a significant higher ITPC for the high amplitude versus low amplitude conditions.

The percentage of simulations that lead to a significant difference in ITPC between the low and high alpha power condition are presented as colorcodes in Fig. 4. As predicted, ITPC values were larger for high power epochs than for low power epochs. Differences are present for SNR values between −40 dB and 0 dB. The absence of a difference for low levels of SNR could be explained by the fact that the SNR is too low to make a phase angle estimation in both conditions. The absence of a difference in ITPC for high levels of SNR could mean that noise does not have an influence on the phase estimation.

The difference in power does not need to be large to find significant differences as seen in Fig. 4. A difference of 0.1 µV (A = 3 µV versus A = 2.9 µV) already led to significant differences, and when the difference was increased to 0.2 µV (A = 3 µV versus A = 2.8 µV) the ITPC difference was stable. The few ITPC differences found when amplitudes were similar (both A = 3 µV) could arise because amplitudes for both conditions contained random jitter which makes it possible that amplitudes differed slightly between conditions.

### The influence of noise on ITPC estimates is present under most levels of phase-locking

In the second simulation the power difference was kept constant, but the analysis was repeated under various amounts of ITPC (please keep in mind that phase angles were identical between conditions that were compared, but repeated with different amounts of ITPC for both conditions). The ITPC was varied by changing the range of possible values of θ (phase angle, see equation 2). The interval of possible phases ranged from [0–0] to [0–2 π] in 11 steps, creating ITPC bins that ranged between 0 and 1. The amplitude of the high and low oscillation where based on power differences in alpha activity between two attentional conditions in one of our own EEG experiments^7^. This resulted in oscillations with amplitudes for the low and high power condition of respectively 2.7 µV and 3.2 µV plus jitter as described in the previous section.

**Figure 5:**
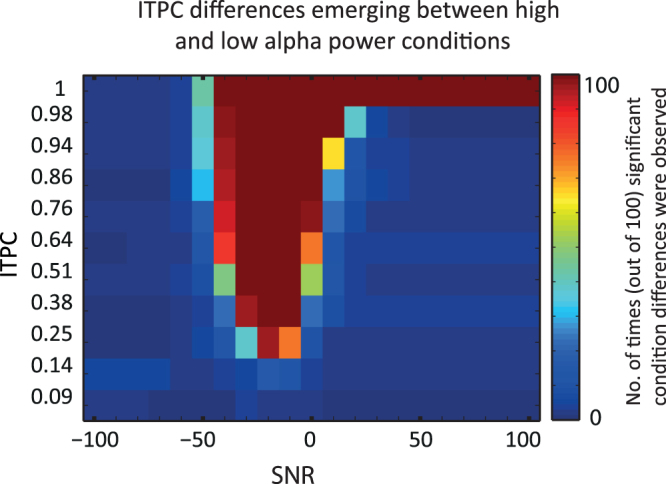
Comparison of ITPC between high and low power epochs under varying phase-locking conditions. Phase angles were identical for the low and high power epochs, but the amount of ITPC was varied between comparisons. Power for the low and high power condition were kept constant, with amplitudes of 2.7 µV and 3.3 µV respectively. Colors represent the percentage of simulations that resulted in a significant higher ITPC estimation for the high versus low alpha power condition.

As evident in Fig. 5 the power induced ITPC difference is present under most levels of phase-locking, and increases as phase-locking among trials increases. Note that the y-axis does not represent the difference in ITPC, but the actual ITPC in both conditions.

## Conclusion

**Figure 6:**
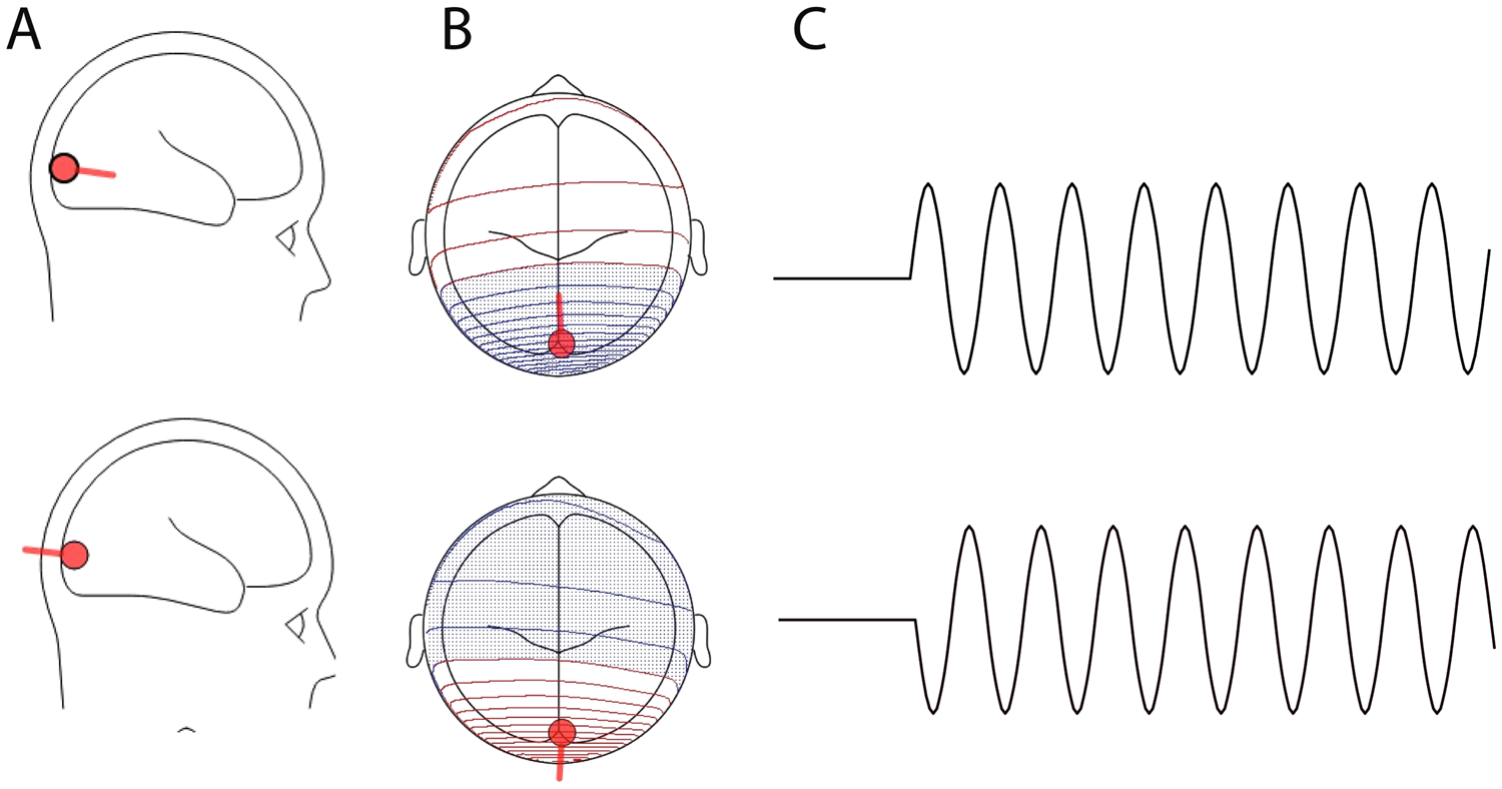
(**A**) EEG signals are believed to mainly reflect the synchronous post-synaptic potentials in the apical dendrites of pyramidal neurons, spatially aligned and perpendicular to the cortical surface. The current flow within the apical dendrite is ‘dipolar’ and as such the summation of the activity of neighboring neurons can be viewed as a dipole. (**B**) Dipoles in the same brain area, but different orientations would create different topographies at the scalp. (**C**) Oscillatory activity generated by dipoles in the same brain region but with different orientations would have different absolute phases when measured at the scalp. This figure was made using BESA Simulator (http://www.besa.de/downloads/besa-simulator/).

Though phase angle perturbations are a subject of many studies in electrophysiological research, conclusive statements are lacking because of the absence of consensus among results. Previous animal work has suggested that attention to a particular sensory stream is able to amplify neuronal responses (to stimuli in that stream) by adjusting the phase of ongoing oscillations to be at their optimal excitatory cycle during anticipated stimulus onset^34^. More recently evidence for this phenomena has been observed in humans using MEG^37^. It should be noted that the reproducibility of this work in human experiments presents a particular challenge. This is due to the fact that variations in the orientation of dipoles generating oscillatory activity would translate in different absolute phases across participants,when the data is epoched relative to an experimental event. A simple illustration of this is in Fig. 6, where we simulated oscillatory activity from the same brain region, but had the generators have different dipole orientations. Such a situation could yield null findings in investigations looking at the relationship of the absolute phase of an ongoing oscillation to an experimental event. One way to circumvent such issues is to examine phase of oscillatory activity from the source reconstructed signal^37,38^. These simulations were done using Besa Simulator a free software for the simulation of electrophysiological activity at the scalp level(http://www.besa.de/downloads/besa-simulator/).

ITPC also gets around this issue, by not looking at the phase-angles, but rather their clustering (i.e uniformity). However, this measure is also prone to some caveats, especially when ITPC is compared between conditions. Simulations show that phase angle estimations can be affected by unwanted factors such as oscillatory power, ERP latency and ERP amplitude. Temporal leakage of evoked responses can increase phase locking among trials. Oscillations with relatively larger amplitudes have a better SNR, which leads to better phase estimations and less variability, hence higher ITPC estimates. Although the influence seems small at first sight, when power or evoked responses systematically differs due to attentional differences or task demands, this could lead to significant differences in ITPC between conditions. Caution should therefore be exercised with interpretation of ITPC differences when a power or ERP difference is present.

There are some steps advocated to reduce the possibility of false discoveries caused by power differences. For example Cohen^39^ suggests finding ITPC differences in the absence of a power difference for certain time points or channels. Furthermore, it is important that power differences are examined using non baseline-corrected frequency data. The simulations indicate that power differences lead to a difference in the ability to estimate phase-angles through differences in SNR. A baseline-correction adjusts the magnitude of the signal (frequency of interest) independent of the noise. This means that baseline-corrected data is not a reflection of the SNR.

The influence of condition specific differences in evoked responses on ITPC is sometimes overlooked. For instance Samaha *et al*.^40^ found an ITPC difference at time of target presentation between correct and incorrect trials. This means that it is not possible to unequivocally discount the possibility that their observation that the phase angles of ongoing alpha activity are modulated by temporal prediction could alternatively be explained by a difference in ERP amplitude and/or latency caused by differences in attentional states between trials. We advocate that any differences in ERP amplitude or latency should be excluded before conclusive statements be made about phase alterations. A way to eliminate the influence of evoked responses completely is to add trials in which a target is expected but not actually presented (as suggested in Van Diepen *et al*.^7^).

An example of a study in which additional analyses were performed to exclude power induced ITPC differences between conditions is by Bonnefond and colleagues^17^. The goal of this experiment was to show increased Inter-Trial Phase-Locking during a strong distractor condition, compared to a weak distractor condition, in a task with temporally predictable stimulus presentation. An ITPC difference was indeed found between the distractor conditions, however, together with a difference in oscillatory power. To rule out the likelihood that the power difference was driving the phase effects, ITPC values were correlated with power. Specifically, the correlation between the ongoing oscillatory power in an individual and the ITPC values was examined, as well as a correlation between differences in ITPC and power between the weak and strong distractor conditions. Also, the Spearman correlation for single-trial power and the deviation of the single-trial phase from the average phase was calculated. Last, trials were divided in quartiles based on alpha power and a trend-analysis was performed on the accompanying ITPC values. The absence of systematic correlations or a (clear) trend was interpreted as evidence that ITPC differences between the conditions could not be explained trivially by differences in oscillatory power.

We propose future work in addition to reporting the p values for the contrast in ITPC between conditions, also report the absolute ITPC values in each condition to allow the readers to assess the size of the effect. Finally, we propose new measures should be developed that can detect phase-adjustment directly. At present only the circumstantial consequence of a phase-adjustment is examined: a difference in ITPC at a certain point in time. Instead, one could search for more direct evidence that the oscillation is speeding up or slowing down in order to reach the optimal phase.

For the simulations a lot of parameters had to be chosen. The ITPC, SNR, oscillatory power and variation in power between epochs, window length of the Hann taper etc. They were chosen based on our own data as much as possible, but other experiments with other parameters can result in different outcomes. In addition, in real EEG data the power and ERP differences might be less systematic, decreasing the probability of an ITPC difference. Nevertheless, the fact that significant differences are found with at least some parameters show that it is a problem that should be considered. Therefore, to attain more conformity among studies examining phase-locking, direct comparisons between conditions should be avoided when systematic differences in power or ERPs are present.

## References

[1] Bauer M, Kennett S, Driver J (2012). Attentional selection of location and modality in vision and touch modulates low-frequency activity in associated sensory cortices. J Neurophysiol.

[2] Haegens S, Handel BF, Jensen O (2011). Top-down controlled alpha band activity in somatosensory areas determines behavioral performance in a discrimination task. J Neurosci.

[3] Haegens S, Nacher V, Luna R, Romo R, Jensen O (2011). alpha-Oscillations in the monkey sensorimotor network influence discrimination performance by rhythmical inhibition of neuronal spiking. Proc Natl Acad Sci USA.

[4] Jokisch D, Jensen O (2007). Modulation of gamma and alpha activity during a working memory task engaging the dorsal or ventral stream. J Neurosci.

[5] Medendorp WP (2007). Oscillatory activity in human parietal and occipital cortex shows hemispheric lateralization and memory effects in a delayed double-step saccade task. Cereb Cortex.

[6] Rihs TA, Michel CM, Thut G (2007). Mechanisms of selective inhibition in visual spatial attention are indexed by alpha-band EEG synchronization. Eur J Neurosci.

[7] van Diepen RM, Cohen MX, Denys D, Mazaheri A (2015). Attention and temporal expectations modulate power, not phase, of ongoing alpha oscillations. J Cogn Neurosci.

[8] Foxe JJ, Simpson GV, Ahlfors SP (1998). Parieto-occipital approximately 10 Hz activity reflects anticipatory state of visual attention mechanisms. Neuroreport.

[9] Foxe JJ, Simpson GV, Ahlfors SP (1998). Parieto-occipital approximately 10 Hz activity reflects anticipatory state of visual attention mechanisms. Neuroreport.

[10] Jensen O, Mazaheri A (2010). Shaping functional architecture by oscillatory alpha activity: gating by inhibition. Front Hum Neurosci.

[11] Klimesch W, Sauseng P, Hanslmayr S (2007). EEG alpha oscillations: the inhibition-timing hypothesis. Brain Res Rev.

[12] Busch NA, Dubois J, VanRullen R (2009). The phase of ongoing EEG oscillations predicts visual perception. J Neurosci.

[13] Mathewson KE, Fabiani M, Gratton G, Beck DM, Lleras A (2010). Rescuing stimuli from invisibility: Inducing a momentary release from visual masking with pre-target entrainment. Cognition.

[14] Mathewson KE (2011). Pulsed out of awareness: EEG alpha oscillations represent a pulsed-inhibition of ongoing cortical processing. Front Psychol.

[15] Mathewson KE (2012). Making waves in the stream of consciousness: entraining oscillations in EEG alpha and fluctuations in visual awareness with rhythmic visual stimulation. J Cogn Neurosci.

[16] Scheeringa R, Mazaheri A, Bojak I, Norris DG, Kleinschmidt A (2011). Modulation of visually evoked cortical FMRI responses by phase of ongoing occipital alpha oscillations. J Neurosci.

[17] Bonnefond M, Jensen O (2012). Alpha oscillations serve to protect working memory maintenance against anticipated distracters. Curr Biol.

[18] Tallon-Baudry C, Bertrand O, Delpuech C, Pernier J (1996). Stimulus specificity of phase-locked and non-phase-locked 40 Hz visual responses in human. J Neurosci.

[19] Di Russo F, Martinez A, Hillyard SA (2003). Source analysis of event-related cortical activity during visuo-spatial attention. Cereb Cortex.

[20] Gomez Gonzalez CM, Clark VP, Fan S, Luck SJ, Hillyard SA (1994). Sources of attention-sensitive visual event-related potentials. Brain Topogr.

[21] Kelly SP, Gomez-Ramirez M, Foxe JJ (2008). Spatial attention modulates initial afferent activity in human primary visual cortex. Cereb Cortex.

[22] Slagter HA, Prinssen S, Reteig LC, Mazaheri A (2016). Facilitation and inhibition in attention: Functional dissociation of pre-stimulus alpha activity, P1, and N1 components. Neuroimage.

[23] Van Voorhis S, Hillyard SA (1977). Visual evoked potentials and selective attention to points in space. Perception & Psychophysics.

[24] Mangun GR, Hillyard SA (1991). Modulations of sensory-evoked brain potentials indicate changes in perceptual processing during visual-spatial priming. J Exp Psychol Hum Percept Perform.

[25] Eason RG (1981). Visual evoked potential correlates of early neural filtering during selective attention. Bulletin of the Psychonomic Society.

[26] Eason RG, Harter MR, White CT (1969). Effects of attention and arousal on visually evoked cortical potentials and reaction time in man. Physiology & Behavior.

[27] Harter, M. R., & Aine, C. J. *Brain mechanisms of visual selective attention*. *Varieties of attention*. 293–321 (1984).

[28] Harter MR, Aine C, Schroeder C (1982). Hemispheric differences in the neural processing of stimulus location and type: effects of selective attention on visual evoked potentials. Neuropsychologia.

[29] Eimer M, Van Velzen J, Gherri E, Press C (2006). Manual response preparation and saccade programming are linked to attention shifts: ERP evidence for covert attentional orienting and spatially specific modulations of visual processing. Brain Res.

[30] Eimer M, van Velzen J (2005). Spatial tuning of tactile attention modulates visual processing within hemifields: an ERP investigation of crossmodal attention. Exp Brain Res.

[31] Baldauf D, Deubel H (2009). Attentional selection of multiple goal positions before rapid hand movement sequences: an event-related potential study. J Cogn Neurosci.

[32] Couperus JW, Mangun GR (2010). Signal enhancement and suppression during visual-spatial selective attention. Brain Res.

[33] Freunberger R (2008). Functional similarities between the P1 component and alpha oscillations. Eur J Neurosci.

[34] Lakatos P, Karmos G, Mehta AD, Ulbert I, Schroeder CE (2008). Entrainment of neuronal oscillations as a mechanism of attentional selection. Science.

[35] Yeung N, Bogacz R, Holroyd CB, Cohen JD (2004). Detection of synchronized oscillations in the electroencephalogram: an evaluation of methods. Psychophysiology.

[36] Mazaheri A, Jensen O (2008). Asymmetric amplitude modulations of brain oscillations generate slow evoked responses. J Neurosci.

[37] Baldauf D, Desimone R (2014). Neural mechanisms of object-based attention. Science.

[38] Berger B, Minarik T, Liuzzi G, Hummel FC, Sauseng P (2014). EEG oscillatory phase-dependent markers of corticospinal excitability in the resting brain. Biomed Res Int.

[39] Cohen, M. X. & M. I. T. CogNet. *Analyzing neural time series data: theory and practice*, (The MIT Press, 2014).

[40] Samaha J, Bauer P, Cimaroli S, Postle BR (2015). Top-down control of the phase of alpha-band oscillations as a mechanism for temporal prediction. Proc Natl Acad Sci USA.

